# Deep learning-based high-throughput phenotyping can drive future discoveries in plant reproductive biology

**DOI:** 10.1007/s00497-021-00407-2

**Published:** 2021-03-16

**Authors:** Cedar Warman, John E. Fowler

**Affiliations:** 1 Department of Botany and Plant Pathology, Oregon State University, Corvallis, OR USA; 2 School of Plant Sciences, University of Arizona, Tucson, AZ USA

**Keywords:** Deep learning, Computer vision, Neural network, Phenotyping, Reproduction

## Abstract

Advances in deep learning are providing a powerful set of image analysis tools that are readily accessible for high-throughput phenotyping applications in plant reproductive biology.

High-throughput phenotyping systems are becoming critical for answering biological questions on a large scale. These systems have historically relied on traditional computer vision techniques. However, neural networks and specifically deep learning are rapidly becoming more powerful and easier to implement. Here, we examine how deep learning can drive phenotyping systems and be used to answer fundamental questions in reproductive biology. We describe previous applications of deep learning in the plant sciences, provide general recommendations for applying these methods to the study of plant reproduction, and present a case study in maize ear phenotyping. Finally, we highlight several examples where deep learning has enabled research that was previously out of reach and discuss the future outlook of these methods.

## Introduction

Linking genotype to phenotype is a powerful method to understand complex biological systems. However, the increasing availability of genotypic data has not been matched by increasing capabilities for high-throughput phenotyping. This is particularly the case in the field of plant reproduction, where relevant biological processes are dependent on subtle cell–cell interactions, often taking place deep within vegetative and reproductive tissues. Until recently, computer vision-based plant phenotyping methods have been dominated by easily observable structures, such as leaves (Arvidsson et al. 2011; Zhang et al. 2012, 2017; Junker et al. 2014; Choudhury et al. 2016; Awlia et al. 2016) and roots (Yazdanbakhsh and Fisahn 2009; Clark et al. 2013; Slovak et al. 2014; Passot et al. 2018; Jiang et al. 2019). These methods have relied on traditional computer vision, which leverages mathematical approaches such as thresholding (Otsu 1979), morphological opening and closing (Haralick et al. 1987), and k-means clustering (Lloyd 1982) to separate the object of interest from the background on a pixel level. These approaches are generally computationally efficient, but struggle when faced with complex backgrounds, closely packed objects, and structures with inconsistent features. Such challenging aspects are often encountered when phenotyping reproductive mutants, limiting the utility of traditional image processing approaches.

Over the past several years, neural network-based deep learning methods have been increasingly applied to address a wide range of biological questions (reviewed in (Ching et al. 2018)). Neural network-based computer vision approaches are fundamentally different from the previous generation of image processing techniques because they rely on large sets of training data to function. Neural networks are composed of multiple layers. For image analysis, the first layer, called the input layer, is made up of the image pixels. The final layer, called the output layer, contains the model’s predictions. Between the input and output layers are one or more hidden layers. Each layer is composed of a number of nodes (or neurons), which perform simple mathematical operations. Nodes are variously connected to adjacent layers and have adjustable parameters that can be trained computationally to accurately predict patterns in the input layer. Data flow from the input layer, through the hidden layers, and finally to the output layer. Neural networks with multiple hidden layers are called deep neural networks.

Neural networks have been used as versatile solutions to many phenotyping challenges. Recent improvements in ease of use and the development of advanced computational resources make deep learning strategies well-situated to drive future discoveries in plant reproductive biology. Here, we examine potential applications of deep learning-based image analysis to overcome phenotyping challenges. We highlight current plant phenotyping systems that take advantage of deep learning, as well as useful deep learning frameworks, guidelines for implementing models, and the future outlook for these methods in plant reproductive biology.

## Opportunities for high-throughput phenotyping in plant reproduction

To date, deep learning approaches in plant phenotyping have been focused on similar targets to traditional computer vision approaches, such as leaves (Ubbens and Stavness 2017; Ziamtsov and Navlakha 2019; Hüther et al. 2020) and roots (Douarre et al. 2018; Wang et al. 2019; Yasrab et al. 2019). Additional applications of deep learning in plants include disease identification (Wang et al. 2017; Polder et al. 2019), inflorescence motion tracking (Gibbs et al. 2019), and fruit shape (Feldmann et al. 2020). These implementations have performed well on images that challenge traditional computer vision approaches, particularly those with complex target objects and backgrounds. However, published tools for phenotyping reproductive tissues are notably scarce.

**Fig. 1:**
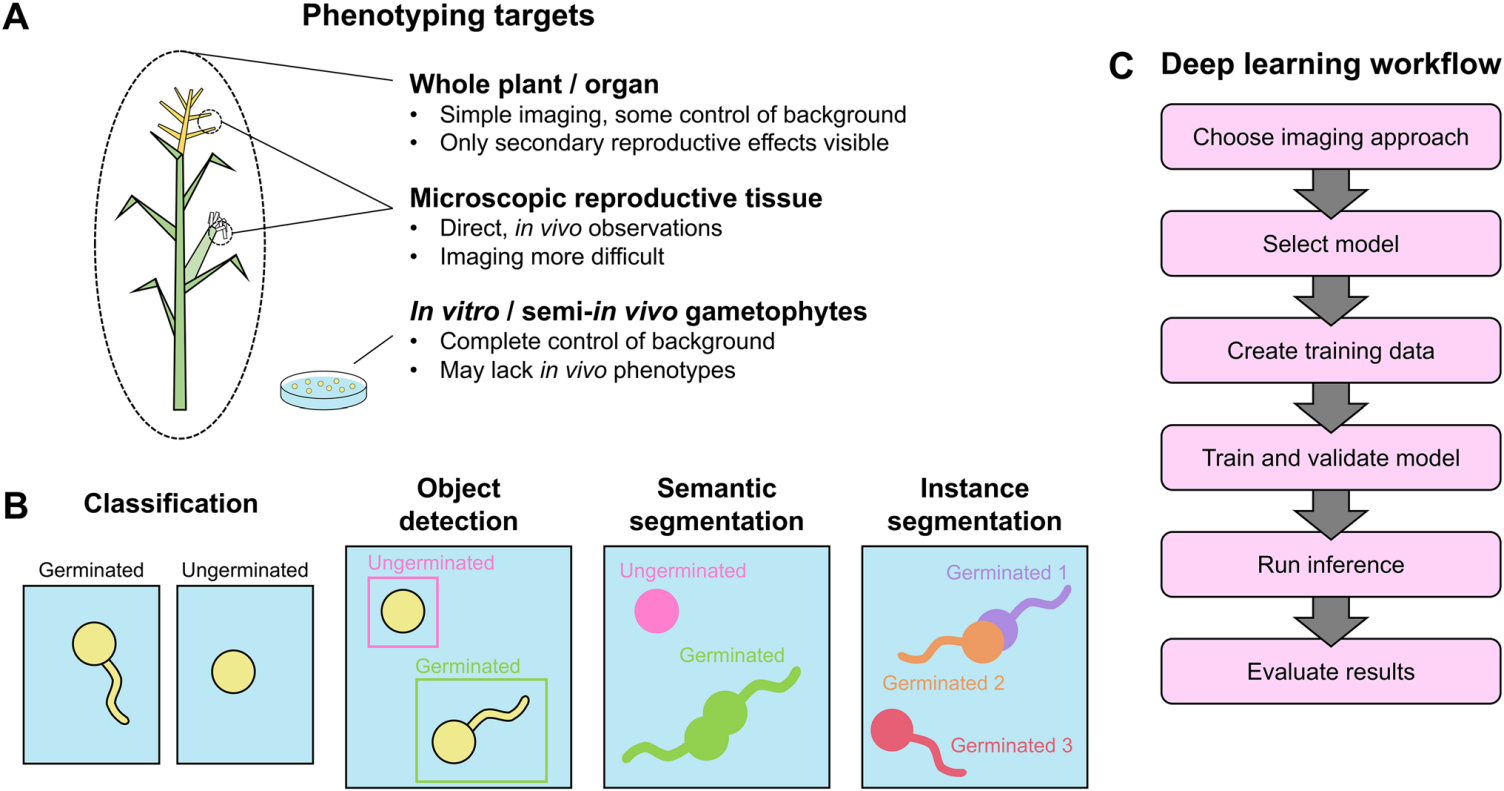
Leveraging deep learning for high-throughput phenotyping in plant reproduction. **a** Potential imaging targets for high-throughput phenotyping of reproductive systems can range from the whole plant to individual organs to microscopic structures, both in vivo and in vitro. **b** General deep learning strategies for image analysis. In classification, the class of each image is described, here germinated versus ungerminated pollen. When a single image contains more than one object, object detection can be used, a method that identifies objects and classes by bounding boxes. Semantic segmentation identifies the class of objects in an image on a pixel level, allowing for the identification of object attributes like shape and area. As with semantic segmentation, instance segmentation identifies pixel classes. In addition, instance segmentation differentiates multiple instances of the same object class that are touching or overlapping. **c** Conceptual steps for implementing deep learning models

Deep learning approaches first require digital representations of the phenotype(s) of interest, generally in the form of images. Conceptually, there are three general targets for plant reproductive phenotyping (Fig. 1a). For the first, whole plant or individual organs such as inflorescences and fruit can be imaged using existing methods. Whole plant or organ imaging is generally simpler than other approaches, but observations are largely limited to secondary effects of reproductive processes, such as abnormalities in fruit shape due to aberrant seed set. Such imaging has been used to track mutant inheritance through kernel phenotypes (Warman et al. 2021), estimate seed yield (Uzal et al. 2018; Khaki et al. 2020), measure fruit characteristics (Hamidinekoo et al. 2020), and classify kernel abortion (Chipindu et al. 2020). Key considerations for whole plant or organ imaging include imaging systems, background selection, and development of methods for discerning the secondary reproductive effects of interest.

A second target for reproductive phenotyping is small reproductive tissues, specifically the male and female gametophytes, the zygote and embryo, and surrounding sporophytic tissues. Phenotyping these tissues are critical because they offer the ability to directly observe reproductive processes in vivo. However, obtaining microscopic images of these tissues on a large scale can be challenging. Often the gametophytes are located deep within other tissues and require special staining or fluorescent markers to visualize structures of interest. In addition, complicated backgrounds can make computer vision approaches challenging, even with the use of deep learning approaches. Preliminary applications of deep learning in such instances have focused on identifying cells from images of plant tissues using segmentation (Wolny et al. 2020). These methods have been used to describe ovule development (Vijayan et al. 2021).

A final type of phenotyping target is gametophytes or zygotes imaged in vitro or semi-in vivo (Higashiyama et al. 2001; Palanivelu and Preuss 2006). These approaches have the potential to allow high-throughput imaging of gametophytes in a controlled environment, making them amenable to deep learning approaches, but may fail to capture some phenotypes that are only observed in vivo. Pollen has been the primary target of these types of approaches, likely because it is relatively easy to isolate compared to the female gametophyte. Previous studies have used deep learning to identify pollen from several different species (Dunker et al. 2020) and differentiate pollen developmental stages (García-Fortea et al. 2020).

## A variety of general purpose deep learning frameworks can be applied to reproductive phenotyping

Deep learning for image analysis can be broadly divided into four categories based on how objects are identified in the image: classification, object detection, semantic segmentation, and instance segmentation (Fig. 1b). In image recognition problems, classification answers what class, or type of object, describes a single image. Classification is possible when individual images contain only one object to be identified, such as single germinated or ungerminated pollen grains (Fig. 1b). If more than one object is present in an image or if the location of the object needs to be determined, an object detection approach may be more suitable. Object detection uses deep learning (often convolutional neural networks or CNNs) to identify both the classes and locations of multiple objects in an image, such as germinated and ungerminated pollen grains, and signifies them by labeled bounding boxes. Some types of experiments, such as shape or area measurements, may require semantic segmentation or instance segmentation. These approaches identify the class of every pixel in an image, outputting a mask over objects in the image that shows not only their location, but their shape. Instance segmentation goes a step farther than semantic segmentation by resolving touching or overlapping objects, and thus also enables the identification of individual objects. Instance segmentation has been used for crop seed phenotyping (Toda et al. 2020), and both methods show potential for measuring other reproductive phenotypes, such as the path of growing pollen tubes or the area of developing embryos. The choice of deep learning strategy will vary by phenotyping task and is important to consider before choosing a model, as models are optimized for specific strategies. An additional consideration is the ease of generating training data, a challenge that is described in more detail in the following section. While all deep learning approaches require training data, creating these data for classification tasks are faster than for object detection and semantic segmentation.

Several programming frameworks are available for implementing deep learning models. Most require some knowledge of the Python or R programming languages, as well as a basic familiarity with command line interfaces and Linux/Unix operating systems. Many machine learning libraries exist, but here we highlight two freely available and open source frameworks that could serve as a useful starting point. Currently, the most popular and versatile library is TensorFlow (Abadi et al. 2016). TensorFlow has a well-developed set of machine learning tools, as well as thorough documentation and an active community of users. Many of the newest deep learning models are implemented in TensorFlow, and it remains at the forefront of machine learning. Taking full advantage of TensorFlow requires a thorough understanding of neural networks and a high degree of coding ability. However, the Keras application programming interface (API) simplifies many of TensorFlow's functions (Chollet and Others 2015) and increases accessibility to researchers who are not primarily focused on machine learning. Several well-established and generalizable TensorFlow methods exist for computer vision tasks, including for object detection and semantic segmentation.

PyTorch (Paszke et al. 2019) is a major alternative to TensorFlow. While the user base and available resources are not as large as with TensorFlow, PyTorch provides some of the same versatile building blocks for neural network construction. PyTorch is tightly integrated with the Python programming language, so researchers with experience in Python may find PyTorch more intuitive to learn than Tensorflow. Similar network architectures can be created with both TensorFlow and PyTorch, so the best library for biological image analysis will depend both on the researcher's preferences and the availability of established methods in each library.

## Implementing deep learning methods

A general workflow for implementing deep learning models can be broken down into a number of conceptual steps (Fig. 1c). Before pursuing deep learning, it is important to ensure the research question is best answered by this approach. Generating large amounts of high-quality training data require a substantial initial time investment. Traditional computer vision methods may be better suited to certain challenges, such as the segmentation of a green plant from a solid background. In addition, hand annotation might be a more efficient method with small sets of images. Deep learning approaches only start to see returns in truly high-throughput cases, with the analysis of hundreds or thousands of images.

If deep learning is well suited to the research question at hand, the first step is to choose which tissues or structures to image, and to develop a process to generate a large quantity of images. Many commercial and academic phenotyping platforms exist for image generation, but the specific challenges of imaging reproductive structures may require the development of new technologies. Alongside image generation, the researcher should choose the category of image analysis (classification, object detection, semantic segmentation, or instance segmentation) and deep learning model that can most effectively answer the research question. For example, counting fertilized ovules may only require object detection, whereas measuring the size of developing seeds could benefit from semantic segmentation.

Next, labeled training images must be generated, and subsequently split into training, validation, and test sets. Training and validation sets allow the model to learn and evaluate itself during the training process, whereas test sets are reserved for unbiased final evaluations of the trained model. A key consideration when training any deep learning model is to avoid overfitting the data. Overfitting results when the model predicts the training data too closely and is consequently not generalizable to new data. Reserving an exclusive test dataset aside from training and validation datasets provides a final check that the model is generalizable beyond the datasets used for its creation. The number of training images required for an accurate model will vary by task, but expect to generate between 100 and 1000 training images, at a minimum. Measuring highly variable phenotypes, such as identifying cell types in mutant ovules, will likely require more training data than measuring relatively straightforward phenotypes, such as counting seeds on a solid background. One commonly used strategy to leverage small amounts of training data is to employ transfer learning (Douarre et al. 2018; Hüther et al. 2020). Transfer learning begins with a well-trained model from a previous deep learning task, often a generalized task such as the identification of common objects, then retrains the top layers of the model with a new training set for the task at hand. Another strategy is to use semi-automated methods to increase the size of training sets, a particularly useful method for semantic segmentation training data (Adams et al. 2020).

After sufficient training data are generated, the model is trained. In deep learning models, training is an iterative process in which parameters of the network's nodes are adjusted to optimize predictions. After each iteration, or epoch, the model adjusts to better predict outputs. Adjustments are typically made to minimize a "loss function," which is a measurement of how accurately the model predicted validation data. Model training time depends on the size of the training dataset and model complexity, and can vary widely, from minutes to days or even longer. The process can be vastly sped up with high performance GPUs, which are available through local or cloud-based computational resources. Many cloud services offer computational resources optimized for deep learning frameworks.

Once a model is trained, predictions, or inference, can be made on experimental datasets. Inference is typically less computationally intensive than model training and can often be run on a consumer grade machine. It is important that the researcher defines how the performance of the model is evaluated throughout the deep learning process. For image classification tasks, performance metrics typically aim to quantify the number of true positive, false positive, false negative, and true negative predictions (often termed the "confusion matrix"). Specific combinations of these quantities include accuracy, precision, recall, and specificity. “Area under the receiver operating characteristic curve” (AUC) is a more thorough formulation of the previous measures. For object detection and semantic segmentation, the accuracy of object location and size predictions can be evaluated using intersection over union (IoU) and mean average precision (mAP). More accessible metrics can also be used. For example, with regression tasks, coefficients of determination comparing model predictions to ground truth are useful metrics, as well as mean absolute deviations.

## Maize ear phenotyping: a case study

**Fig. 2:**
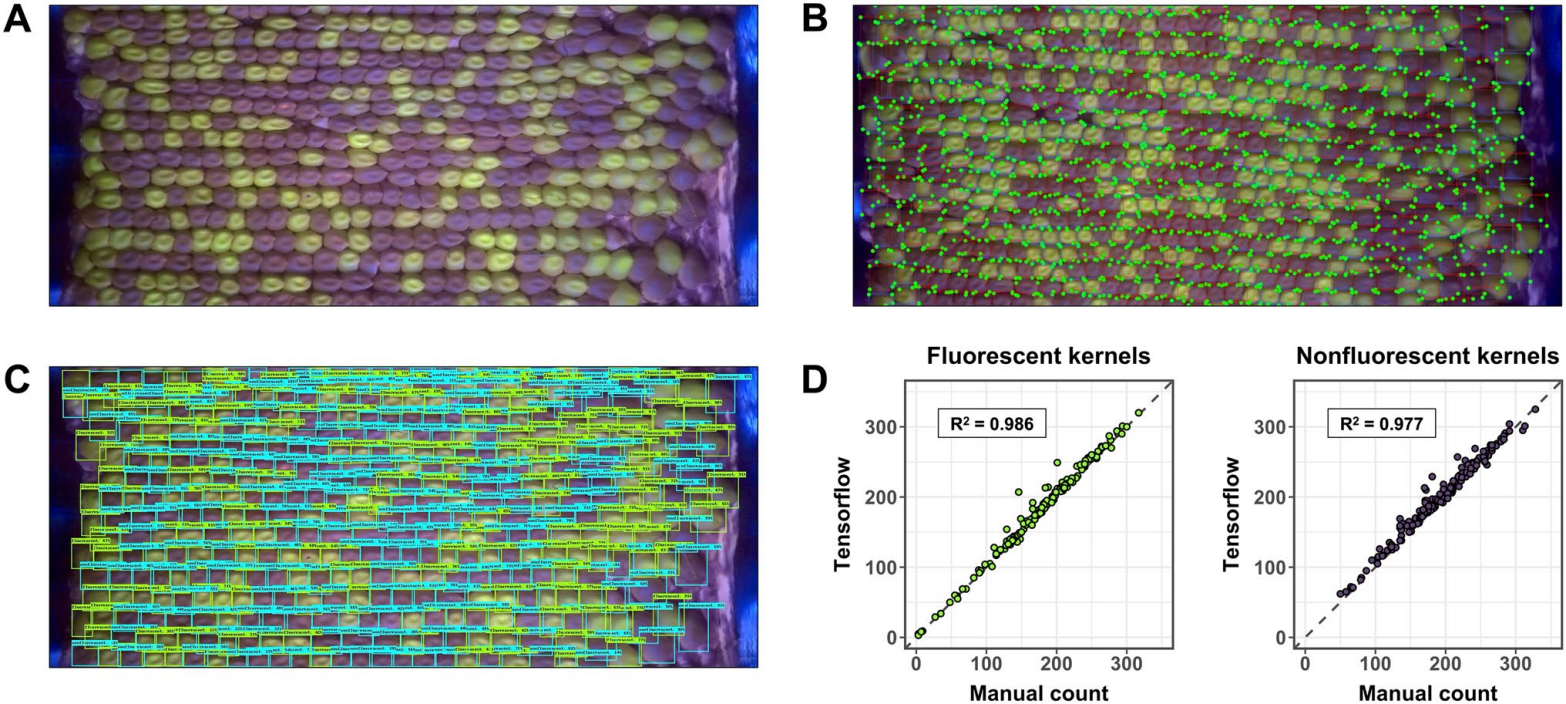
Maize ear phenotyping as an example deep learning workflow. **a** First, a rotational scanning system creates a flat projection of the surface of an ear. Fluorescent kernel markers are visible in this projection, signifying the presence of a genetically engineered transposable element insertion in a gene of interest. The ratio of fluorescent (mutant) kernels to non-fluorescent (wild-type) kernels can be tracked to screen for non-Mendelian inheritance of the mutant alleles. **b** Next, a training set of 300 projections with manually assigned bounding boxes labeling each kernel (corners marked by green circles) was generated. A transfer learning approach and the Tensorflow Object Detection API was then used to create a model based on the training dataset. **c** Model inference on the independent test set generates bounding boxes predicting the locations of the objects of interest in the image. Blue boxes signify non-fluorescent kernels and green boxes signify fluorescent kernels. **d** A comparison between model predictions and manual counts for fluorescent and non-fluorescent kernels (160 ear projections) was used to validate the model

Our recent work to create a high-throughput phenotyping system for maize ears can serve as a practical example of one implementation of deep learning methods for plant reproductive phenotyping (Warman et al. 2021). Our goal was to track changes in Mendelian inheritance caused by mutations in genes highly expressed in the male gametophyte. To do so, we used a collection of 56 GFP-tagged transposable element insertion mutants (Li et al. 2013; Warman et al. 2020). Kernels carrying mutant alleles express the Green Fluorescent Protein (GFP), allowing quantification of mutant transmission by counting fluorescent kernels. To facilitate high-throughput phenotyping, our method targeted the maize ear as a proxy, as functional defects in the male gametophyte will result in altered transmission frequencies. We developed a custom rotational scanning system to capture images of the ear for downstream analysis (Fig. 2a). We chose to use a deep learning approach to quantify kernels because traditional computer vision methods failed to overcome variations in our images, such as kernel shape and fluorescence intensity. For our deep learning model, we used the TensorFlow Object Detection API (Huang et al. 2016) implementation of Faster R-CNN with Inception Resnet v2 (Ren et al. 2015; Szegedy et al. 2016). This approach combined a powerful model with a simple API. We chose object detection because our primary goal was to count kernels to track mutant transmission. Segmentation-based approaches would have provided more detailed descriptions of each kernel, but bounding boxes gave us the information we needed, while minimizing the time required to produce training data. For training the model, we generated 300 images annotated with bounding boxes (Fig. 2b), evenly distributed across ears generated in two field seasons. Each image took approximately 20 min to label, for a total of 100 h of labor to create the entire dataset.

After image annotation, we split the dataset into training and validation sets. We used a transfer learning approach, taking advantage of a network pre-trained on the Collection of Common Objects (COCO) dataset, which consists of ~ 200,000 labeled images of everyday objects. Transfer learning reduced both the amount of training data that was required, as well as the time required to train our model. The model was trained on an Nvidia V100 GPU for approximately one hour. After the model was trained, we performed inference on the testing set, a set of 320 manually counted images that were not used in the training process (Fig. 2c). We measured mAP, as well as calculated linear regressions and mean absolute deviations comparing hand counts to model predictions. The outcome was a highly accurate model, with adjusted *R*^2^ for kernel counts between 0.96 and 0.98, depending on field season and phenotypic marker (Fig. 2d).

The main challenges to implementing deep learning methods were imaging and generating the training data. We found that using consistent imaging conditions (e.g., the same camera) was important, as networks trained on data from one camera were not easily transferable to data collected on another camera. In addition, the large number of kernels present in each image reached the limits of our GPU's memory capacity, requiring a strategy of image subdivision and recombination. Ultimately, deep learning methods enabled us to both (1) survey a larger number of ears than would have been possible by manual counting, leading to the discovery of new mutants with quantitative phenotypic effects; and (2) generate larger datasets for each allele, enabling a more accurate estimation of transmission rate and increased statistical power to identify significant effects.

## The future of deep learning in reproductive phenotyping

Deep learning is a rapidly advancing field. Many deep learning models are general purpose, allowing advancements in unrelated fields to be rapidly applied to specific cases like plant reproduction phenotyping. A thorough understanding of general purpose machine learning libraries like TensorFlow and PyTorch will enable biologists to take advantage of new research in image analysis. While classification and object detection models are relatively mature, semantic segmentation models are an area of active research. These models will gradually improve, allowing for more reliable identification of complex objects in noisy images, such as pollen tubes growing down the pistil or ovules at fertilization.

Large, high-quality training datasets improve the accuracy and versatility of models. Generating training datasets remains a major barrier to implementing these models. However, various methods of data augmentation can help researchers make the most out of available data. Traditional image augmentations include transformations like random cropping, horizontal and vertical flips, hue adjustments, and the addition of random noise. Recent work has developed new methods for increasing the size of training sets. One method, called "domain randomization," has successfully been used in wheat to generate artificial training data by semi-randomly layering cropped images of single wheat seeds to simulate a large number of seeds on a flat surface (Toda et al. 2020). Domain randomization has also been used in Arabidopsis to generate artificial training data for leaf segmentation (Ward et al. 2018). Another method takes advantage of generative adversarial networks, or GANs (Zhu et al. 2018). GANs are a type of machine learning that uses two neural networks to compete against each other to generate realistic data. One network generates data (in this case images), while the other network evaluates the data to determine which examples are real and which are artificial. The network learns from these comparisons and can use this knowledge to create novel images that have the characteristics of the input set. These types of networks have been used to improve accuracy by increasing the size of training datasets.

Another potential future direction is an expansion of what constitutes a phenotype. Historically, phenotypes have included simple concepts like malformed or undeveloped structures, growth defects, or color changes, as well as quantitative effects, such as natural variation in fertilization success in populations across a species’ geographic distribution. Neural networks allow for the measurement of more subtle, multidimensional phenotypes, such as abstractions for how images of plants change in response to treatments like drought or heat. An application of this concept, called latent space phenotyping (LSP), shows strong potential at characterizing changes in images of plants unrecognizable by the human eye (Gage et al. 2019; Ubbens et al. 2020). This approach compares sets of images from treated and control samples by using a neural network to create compressed representations of visual characteristics of the images, termed latent space. This process does not require labeled data beyond experimental and control sets, relying on the neural network to determine multidimensional phenotypes characteristic of the experimental set. These LSPs can then be interpreted using additional networks, ultimately uncovering complex phenotypes that, for example, can be used for genome-wide association studies (GWAS). LSP circumvents the need for hand-labeled training sets and also has the potential to incorporate temporal changes into such phenotypes (Taghavi Namin et al. 2018). Additional methods can take more explicit approaches, such as multitask learning, which optimizes networks based on several predetermined phenotypes simultaneously (Dobrescu et al. 2020).

Implementing deep learning methods for phenotyping can be intimidating, as the time and effort necessary to develop a successful model is significant, and therefore not appropriate for all experiments. However, the power and versatility of these methods make them particularly useful for high-throughput phenotyping, allowing for increased throughput, precision, and the measurement of complex phenotypes that may be difficult to describe using more traditional methods. As their use spreads, deep learning methods will become more accessible, making now an ideal time for applications in high-throughput phenotyping in plant reproductive biology.

## Definitions sidebar: deep learning terminology

*Neural network* A biologically inspired computing system that transforms input data (in this case images) into outputs (in this case predictions). Neural networks contain nodes, inspired by biological neurons, that are arranged in multiple layers, or collections of nodes. In its most basic form, each node can aggregate data from previous nodes, transform the data using trainable weights, and send the data to nodes in the following layer. Deep learning is a subset of machine learning that combines many-layered neural networks with large amounts of training data to enable predictions from heterogeneous datasets.

*Classification* The prediction of a label (also known as a class) from input data. For a single image input, a single class would be predicted, such as germinated or ungerminated pollen.

*Object detection* The detection and localization of objects of one or more classes in an image. Unlike classification, object detection enables multiple objects to be identified in a single image.

*Semantic segmentation* Classification at the pixel level. Semantic segmentation enables the identification of objects in greater detail than object detection.

*Instance segmentation* Classification at the pixel level, with the addition of differentiation of touching or overlapping instances of one or more classes.

*Convolutional neural network (CNN)* A neural network containing convolutional layers. Convolutional layers aggregate image data by moving a filter across the image and summarizing pixel values using mathematical operations. Convolutional layers allow the network to learn patterns in pixel values across the entire image.

*Training, validation, and test sets* Data used to train a neural network model are typically divided into training, validation, and test sets. The training set, usually the largest portion of the input data, is used to train the model. During each round of training, the training set is used to adjust the weights of individual nodes to improve the model’s accuracy. The validation set is used in the training process to evaluate the model's performance after each training round. Separate training and validation sets are used to prevent overfitting, a situation that can arise from the model learning the patterns of the training dataset too specifically and lacking generalizability. The test set is used after training to evaluate the model. Because the model is not exposed to the test set until after training, evaluating the model with the test set provides an unbiased method to test the model’s performance.

*Loss function* During the training process, neural networks are evaluated using a loss function. Loss functions quantify the accuracy of the network’s predictions. Neural networks iteratively optimize the loss function during training using a process called stochastic gradient descent.

## Definitions sidebar: evaluating model performance

*Confusion matrix* Table used to evaluate binary classification models, based on class predictions and class ground truth.
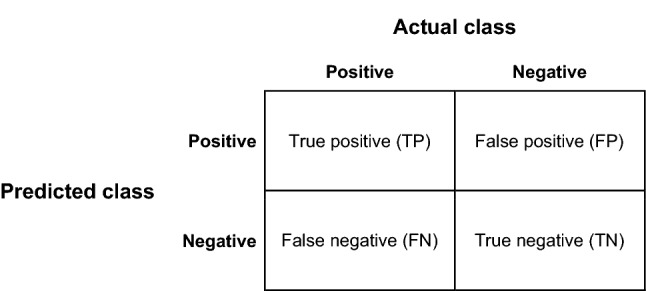


*Accuracy* $\frac{{{\text{TP}} + {\text{TN}}}}{{{\text{TP }} + {\text{TN}} + {\text{FP}} + {\text{FN}}}}$ A measure of how often the model predicted the correct class. Accuracy is a poor measure of model performance because models with higher accuracy do not always have more predictive power than models with lower accuracy.

*Precision* $\frac{{{\text{TP}}}}{{{\text{TP}} + {\text{FP}}}}$ Precision measures the fraction of correct positives over the total number of positive predictions. This metric is useful for measuring a model's performance when true positives are uncommon, and the cost of a false positive is high.

*Recall* $\frac{{{\text{TP}}}}{{{\text{TP}} + {\text{FN}}}}$ Recall measures the proportion of positives that were correctly identified by the model. Precision and recall are often reported together, as an increase in recall is typically associated with a decrease in precision.

*Specificity* $\frac{{{\text{TN}}}}{{{\text{TN}} + {\text{FP}}}}$ Specificity measures the proportion of negatives that were correctly identified by the model.

*Area under the receiver operating characteristic curve (AUC)* AUC is a measure of a binary classification model’s performance that summarizes true positive and false positive rates. An AUC of 1 represents perfect separation of two classes, whereas a model with an AUC of 0.5 performs no better than random.

*Intersection over union (IoU)* IoU is a method to evaluate predictions in object detection and semantic segmentation models. The method compares predicted bounding boxes or masks to ground truth annotations. Intersection describes the overlapping area between the prediction and the ground truth. Union describes the total area covered by both the prediction and the ground truth. If the prediction and ground truth perfectly overlap, IoU is 1, whereas incomplete overlap generates values less than 1.

*Mean average precision (mAP)* mAP is a performance metric often used to evaluate object detection or semantic segmentation models. This method summarizes a precision recall curve (similar to AUC) across all classes and at various IoU thresholds.

### Author contribution statements

CW conceptualized the article and performed literature review. CW and JF wrote and revised the manuscript. CW and JF read and approved the manuscript.
